# Donor Age and Red Cell Age Contribute to the Variance in Lorrca Indices in Healthy Donors for Next Generation Ektacytometry: A Pilot Study

**DOI:** 10.3389/fphys.2021.639722

**Published:** 2021-03-02

**Authors:** Ankie M. van Cromvoirt, Simone Fenk, Ario Sadafi, Elizaveta V. Melnikova, Denis A. Lagutkin, Kuntal Dey, Irina Yu. Petrushanko, Inga Hegemann, Jeroen S. Goede, Anna Bogdanova

**Affiliations:** ^1^Red Blood Cell Research Group, Vetsuisse Faculty, Institute of Veterinary Physiology, University of Zurich, Zurich, Switzerland; ^2^Helmholtz Zentrum München – German Research Center for Environmental Health, Munich, Germany; ^3^Computer Aided Medical Procedures, Technische Universität München, Munich, Germany; ^4^Engelhardt Institute of Molecular Biology, Russian Academy of Sciences, Moscow, Russia; ^5^Department of Medical Oncology and Hematology, University Hospital and University of Zurich, Zurich, Switzerland; ^6^Division of Oncology and Hematology, Kantonsspital Winterthur, Winterthur, Switzerland; ^7^Zurich Center for Integrative Human Physiology (ZIHP), University of Zurich, Zurich, Switzerland

**Keywords:** red blood cells (erythrocytes), deformability, ektacytometry, aging, hydration status, osmotic fragility of RBCs

## Abstract

The ability of red blood cells (RBCs) to transport gases, their lifespan as well as their rheological properties invariably depend on the deformability, hydration, and membrane stability of these cells, which can be measured by Laser optical rotational red cell analyser (Lorrca® Maxsis, RR Mechatronics). The osmoscan mode of Lorrca is currently used in diagnosis of rare anemias in clinical laboratories. However, a broad range of normal values for healthy subjects reduces the sensitivity of this method for diagnosis of mild disease phenotype. In this pilot study, we explored the impact of age and gender of 45 healthy donors, as well as RBC age on the Lorrca indices. Whereas gender did not affect the Lorrca indices in our study, the age donors had a profound effect on the O_hyper parameter. To study the impact of RBC age on the osmoscan parameters, we have isolated low (L)-, medium (M)-, or high (H)- density fractions enriched with young, mature, and senescent RBCs, respectively, and evaluated the influence of RBC age-related properties, such as density, morphology, and redox state, on the osmoscan indices. As before, O_hyper was the most sensitive parameter, dropping markedly with an increase in RBC density and age. Senescence was associated with a decrease in deformability (EI_max) and tolerability to low and high osmolatites (Area). L-fraction was enriched with reticulocytes and cells with high projected area and EMA staining, but also contained a small number of cells small in projected area and most likely, terminally senescent. L-fraction was on average slightly less deformable than mature cells. The cells from the L-fraction produced more oxidants and NO than all other fractions. However, RBCs from the L-fraction contained maximal levels of reduced thiols compared to other fractions. Our study suggests that reference values for O_hyper should be age-stratified, and, most probably, corrected for the average RBC age. Further multi-center study is required to validate these suggestions before implementing them into clinical practice.

## Introduction

**Figure 1:**
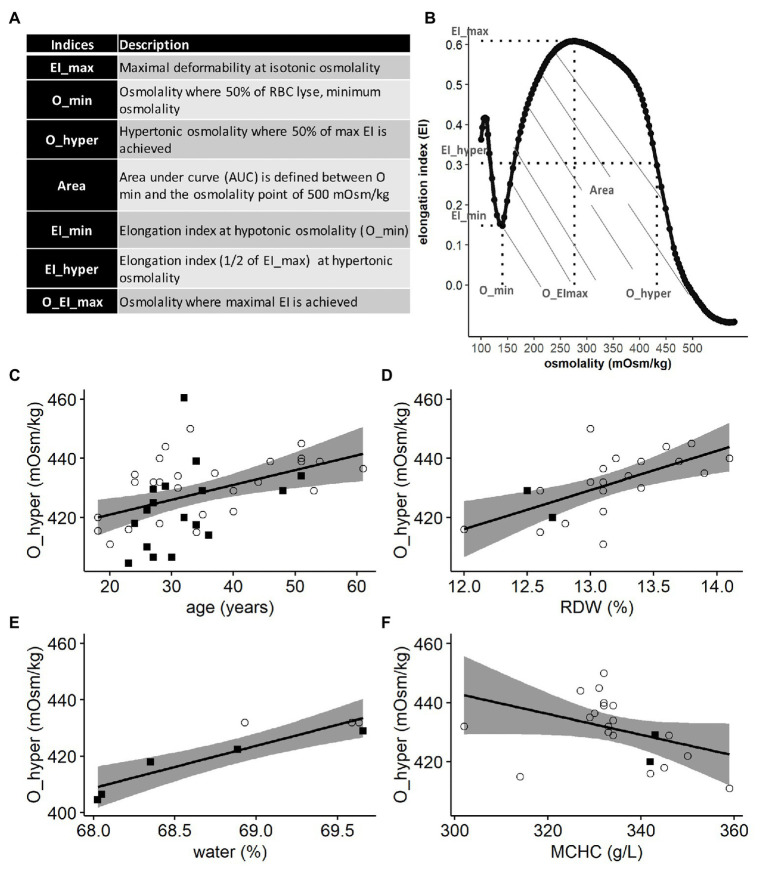
Lorrca indices given by the osmoscan mode. The osmoscan mode on Lorrca gives 7 Lorrca indices, which are listed in the table **(A)** and schematically depicted in the osmoscan curve **(B)** EI: elongation index and AUC: area under the curve. **(C–F)** Associations of Lorrca indices with RBC indices, linear model (lm). **(C)** Association between O_hyper and age (*p* = 0.005), gender is not significant (*p* = 0.08), *N* = 45. **(D)** Association between O_hyper and RDW (*p* = 0.0017), *N* = 22 **(E)** Confirmation that O_hyper is associated with intracellular water measured by ratio dry/wet weight of RBC (*p* = 0.0017), *N* = 8 **(F)** A trend toward an association between O_hyper and MCHC (*p* = 0.06), age is significant in the model (*p* = 0.041), *N* = 22. Females (circles) and males (squares), RDW: red cell distribution width and MCHC: mean corpuscular hemoglobin concentration.

Defective deformability of red blood cells (RBCs) has long been recognized as a hallmark of hemolytic anemias, and several techniques were established to measure it (LaCelle, 1970; Tillmann, 1986). Filtration through the filters and columns, sucking of a cell into the pipette and morphometry of RBCs in flow, are still used to assess deformability (Linderkamp et al., 1986; Relevy et al., 2008; Huisjes et al., 2018). Developed by Bessis and Mohandas in 1975 (Bessis, 1977), the viscodiffractometry or ektacytometry was designed to detect the ability of cells to elongate in response to shear stress over a broad range of extracellular osmolalities. The degree of deformation of RBCs in response to shear stress in Couette flow is visualized as an elongation in light diffraction pattern. Elongation index (EI) was then expressed in units reflecting the ratio between the shortest to the longest diameter for the diffraction image (Mohandas et al., 1980a). The ability to deform was then assessed for the cells with optimal water content, as well as in osmotically swollen and dehydrated RBCs and expressed as the corresponding elongation indices: EI_max, EI_min, and EI_hyper. Stability of RBCs to shear was defined by the minimal (O_min) and maximal (O_hyper) osmolalities the cells were able to tolerate when sheared. Overall stability of RBCs to shear stress over the whole range of tolerated osmolalities was defined as the area under the curve (Area) in which EI was plotted as a function of osmolality (Nemeth et al., 2015). A representative osmoscan obtained by ektacytometry is shown in Figure 1B. Ektacytometry has established itself as useful and reliable test in the diagnosis of hereditary hemolytic anemias associated with mutations in cytoskeletal proteins, ion channels, metabolic enzymes, and hemoglobin (Hb) as each pathology results in a characteristic shift in the osmoscan curve (Mohandas et al., 1980b).

The first generation ektacytometer produced by Technicon relied on self-made consumables and a more flexible, but less user-friendly interface (Clark et al., 1983). It used a dextran solution as diluting agent for the blood sample to reach the viscosity of 10 cP. In a second-generation device, Lorrca MaxSis (RR Mechatronics), a polyvinylpyrrolidone (PVP)-based diluting reagent is used instead of dextrane. As the viscosity of PVP is higher than that of dextrane (30 vs. 21 cP), lower rotation speed is applied to generate Couette flow in which RBCs are exposed to identical shear stress. Nemeth et al. (2015) showed that the osmoscan readouts could not be obtained at shear stress of 1 Pa and remained noisy up to a shear of 20 Pa. The least noisy readouts were obtained at 30 Pa. Choice of this supraphysiological shear stress levels for both machines had an impact of maximal deformability EImax, which was shown to increase with a growing shear stress, and on the ionic strength O_EI_max at which maximal deformability is reached (Heo et al., 2015; Nemeth et al., 2015).

Initial characterization of parameters, impacting osmoscan indices, was extensively performed using the Technicon devices. Among those are decrease in deformability of RBC with cell aging (Lutz et al., 1992; Bosch et al., 1994), variance in surface-to-volume ratio, mean corpuscular Hb concentration (MCHC), metabolic state, and intracellular Ca^2+^ (Mohandas et al., 1980a,b, 1981; Clark et al., 1981; Waugh et al., 1992). At the same time, the blood of patients with rare anemias was investigated (Clark et al., 1980), and as the Technicon ektacytometers were replaced by the second generation Lorrca MaxSis devices produced by RR Mechatronics, scrupulous comparison of the readouts obtained by both types of devices was performed for blood of patients with hereditary hemolytic anemia. (Da Costa et al., 2016; Lazarova et al., 2017; Ballas et al., 2018; Ciepiela, 2018; Llaudet-Planas et al., 2018; Zaninoni et al., 2018; Kaestner and Bianchi, 2020; Zaidi et al., 2020; Zama et al., 2020). The number of reports on the clinical applications of the device for the assessment of the changes in rheology for human patients is steadily growing. However, understanding why patients shift lies in the lack of understanding what causes the variance in Lorrca indices in healthy controls.

In the present pilot study, we were searching for the factors that cause variance in readouts of Lorrca MaxSis indices for blood of healthy donors. We focused on age and gender of donors as well as age of RBCs that in turn reflects turnover that is increased in patients with hereditary hemolytic anemia. Based on the outcome of this study, we planned to invite our colleagues, who use Lorrca MaxSis at the clinical laboratories to participate in a follow-up multi-center study in which the improved protocols for choosing the “healthy reference range parameters” will be developed for the osmoscan assay.

## Materials and Methods

### Blood Samples

Heparinised venous blood samples were obtained from 45 healthy donors with 28 women (18–61 years old) and 17 men (23–51 years old) by qualified skillful personnel. Information on the study participants and RBC indices are presented in Table 1. The percentage of hypochromic RBC was below 5% for all subjects, which indicates the absence of iron deficiency. In agreement with Helsinki convention, healthy study subjects gave informed consent. Blood samples were provided by the Clinical Laboratory of Cantonal Hospital Winterthur, Switzerland, which organized the samples for the calibration of blood analyzers including Lorrca MaxSis. Clinical blood analysis was performed using ADVIA Blood analyser (Siemens) and CO-oximetry performed using blood gas analyser ABL825 FLEX (Radiometer).

**Table 1:** Characteristics of healthy controls enrolled.

	*N*	Age range (years)	Hemoglobin (g/L)	Hematocrit (%)
Female	28	18–61	136.6 ± 7.67	41.4 ± 2.69
Male	17	23–51	151.5 ± 8.92	46.2 ± 3.07
	***N***	**MCV (fL)**	**MCHC (g/L)**	**RDW (%)**
	23	89.6 ± 2.58	334 ± 11.9	13.2 ± 0.51

Data are presented as mean ± SD. MCV: mean cellular volume; MCHC: mean corpuscular hemoglobin concentration; and RDW: red cell distribution width.

### Red Blood Cell Density Separation

Fractionation of RBCs according to their density on isotonic (by calculation) Percoll density gradient was used to obtain RBC fractions enriched with young, mature, or senescent RBCs. Ninety percent isotonic Percoll solution (GE Healthcare, Little Chalfont, Buckinghamshire, United Kingdom; density 1.13 g/ml) diluted with plasma-like medium. Plasma-like medium [(mM) 140 NaCl, 4 KCl, 0.75 MgSO_4_, 10 glucose, 0.015 ZnCl_2_, 0.2 alanine, 0.2 glutamate-Na, 0.2 glycine, 0.1 arginine-HCl, 0.6 glutamine, 20 HEPES-imidazole, and pH 7.4 at RT] supplemented with 0.1% bovine serum albumin was prepared for each blood sample (Makhro et al., 2017). One milliliter of blood was mixed with 13 ml of isotonic Percoll and centrifugation was performed at 20,000×*g* for 30 min at 30–34°C (Sorvall RC 5C plus, rotor SM-24).

Low (L)-, medium (M)-, and high (H)- density fractions were harvested, and RBCs were washed three times [2,000×*g*, 5 min, room temperature (RT)] and resuspended in plasma-like medium for further characterization. In addition, combined fractions from Percoll were taken and washed for further analysis.

### Membrane Band 4.1 a/b Ratio

Membranes were isolated from the cells forming L-, M-, and H-fraction as well as from whole blood-derived erythrocytes by the following protocol of Jackson et al. (2007) with modifications. Erythrocytes were hemolyzed by adding 1 volume of cells to at least 15 volumes of ice-cold PBS buffer containing 0.03–0.04% saponin. After adding the buffer, the cells were kept on ice for 5 min and then sedimented by centrifuging at 14,000 *g* for 20 min. The hemoglobin-free membranes (ghosts) were collected leaving all the debris in the center of the pellet, and washed two times in PBS (14,000×*g* for 10 min). This gave a clean preparation of the RBC membrane. For the determination of 4.1a/b ratio, proteins were separated on the SDS PAGE gel with the subsequent visualization using Coomassie blue staining. Images of the gels were taken using a CoolSNAPcf camera (Photometrics, Tucson, AZ, United States) equipped with Sigma 50 mm 1:2.8 DC MACROD objective (Hama GmbH & Co KG, Monheim, Germany). Densitometric analysis was performed using Image studio lite software (LI-COR Biosciences).

### Water Content by Gravimetry

The fresh blood sample (200 μl) was washed four times in Mg(NO_3_)_2_-imidazole buffer [0.5 M Mg(NO_3_)_2_, 0.1 M imidazole, and pH 7.4]. Supernatant and buffy coat were removed, and the wet RBC pellet was weighed. The pellet was dried for 3 days at 80°C before the dry weight was determined. The ratio of dry weight to wet weight was calculated to get the percentage of water in the native RBC.

### Ektacytometry

RBC deformability was assessed on a Laser Optical Rotational Red Cell Analyzer (Lorrca MaxSis, RR Mechatronics). The EI of RBC was measured within the extracellular osmolality range of 0–500 mOsm/kg at a constant shear stress (30 Pa) and temperature (37°C). Before the measurement, the fresh blood or RBC suspension prepared from the L-, M-, and H-fraction (200 μL) was diluted in 5 ml of isotonic PVP solution (RR Mechatronics) and mixed carefully. Analysis of the osmoscan curve was performed, and the following set of Lorrca indices obtained: the minimal osmolality (O_min), where 50% of RBC are lysed (Clark et al., 1983) in a hypoosmotic environment and its according minimal elongation index (EI_min), the maximal elongation index (EI_max) at optimal osmolality (O_EI_max), the hyperosmotic osmolality (O_hyper), where half of the maximal elongation index (EI_hyper) is reached, and the area under the osmoscan curve (Area; Figures 1A,B).

### Flow Cytometry

The following parameters were detected using flow cytometry (Gallios, Beckman Coulter or LSRFortessa, Becton Dickinson): geometric mean of forward scatter (FS) and side scatter (SS) and their standard deviations, RBC membrane surface area [eosin 5-maleimide (EMA) staining], total and immature reticulocyte counts (RNA staining and CD71 staining, respectively), reduced protein- and non-protein thiol levels (monobromobimane, mBBr, and ThiolTracker Violet), free radical production (dihydrorhodamine 123, DHR), and NO (N_2_O_3_) levels (4-amino-5-methylamino-2',7'-difluorofluorescein diacetate, DAF-FM DA).

Two microliters of blood was incubated in plasma-like medium containing 0.5 mg/ml EMA staining (Merck). After 1 h of incubation in darkness, the excess of EMA was washed away in plasma like medium at 2,500×g for 30 s, triple washing and cells were resuspended in 1 ml of plasma-like medium. Intracellular thiols, including glutathione, N-acetylcysteine, mercaptopurine, and SH-groups of proteins, were measured with mBBr (ThermoFisher). Loading of cells with mBBr was performed in plasma-like medium supplemented with 2 μl of blood. One microliter of blood was resuspended in Thiazole Orange (BD Reti-CountTM) to measure the presence of RNA. ThiolTracker Violet (Invitrogen, ThermoFisher; Mandavilli and Janes, 2010) was used to monitor reduced thiols of intracellular glutathione. DHR (Invitrogen, ThermoFisher) staining reflected production of H_2_O_2_ or ONOO^−^ (Henderson and Chappell, 1993; Crow, 1997), and DAF-FM DA (Invitrogen, ThermoFisher) was applied to assess nitric oxide production by recording the formation of N_2_O_3_ in RBCs (Balcerczyk et al., 2005) of L-, M-, and H-fractions. Suspension of RBCs isolated from L-, M-, and H-fractions were containing equal RBC density (cell counts per volume of suspension). To stain the cells, 5 μl of suspensions were added to 100 μl plasma-like medium and pre-loaded with either 20 μM ThiolTracker Violet or 7.5 μM DHR or 5 μM DAF-FM DA for 30 min at 37°C in the darkness. Thereafter, RBC fluorescence was assessed. Data analyses were performed using Kaluza (Beckman Coulter) or FlowJo (Becton Dickinson) software.

### Microscopy

One microliter of L-, M-, or H-fraction was resuspended in 1 ml of plasma-like buffer supplemented with 0.1% bovine serum albumin. The cell suspension was pipetted in a glass bottom microscopy chamber. RBCs were allowed to settle down for 5–10 min and then images were taken by an inverted microscope (Zeiss Axiovert, 100x objective, Axiocam). The images were analyzed by a deep neural network called Mask R-CNN for single cell segmentation. These segmentation masks were used for further cell by cell morphology analysis (He et al., 2020). Details of the analysis algorithm are described elsewhere (Sadafi et al., 2019).

### Statistical Analysis

Statistical analysis was performed in R (R version 4.0.2 (20202–06-22)). Shapiro-Wilk test was used for checking if the data were normally distributed. Subsequently, a parametric (student’s *t*-test) or non-parametric test (Wilcoxon test) was used to compare the different groups. For detection of associations between Lorrca indices with other parameters, ANOVA and linear models (lm) were used. Specific details are included in the figure legends and power calculations can be found in the supplementary (Supplementary Table S1).

## Results

Osmoscan curves were obtained for all 45 study participants and the resulting indices (Figures 1A,B) were obtained for whole blood and for the L-, M-, and H-density fractions obtained after centrifugation in self-forming continuous Percoll density gradient. We have then analyzed the Lorrca indices for associations with clinical blood parameters (RBC indices), density, morphology of living cells, and redox state.

### Lorrca Indices and Age of Patients

Of all Lorrca indices, O_hyper was the only one showing significant association with the age of the donor (Figure 1C), independent of the gender (Supplementary Figure S1). Increase in O_hyper was positively associated with the RBC distribution width (RDW), an upregulation, which is a nonselective sign of distress (Figure 1D). Being a marker of RBC hydration state, O_hyper showed a strong association with the intracellular water content (measured manually using gravimetry; Figure 1E). However, O_hyper was not associated with mean corpuscular hemoglobin concentration (MCHC) measured by ADVIA2120i blood analyzer (Figure 1F).

### Lorrca Indices for RBCs Forming L-, M-, and H-Fractions

**Figure 2:**
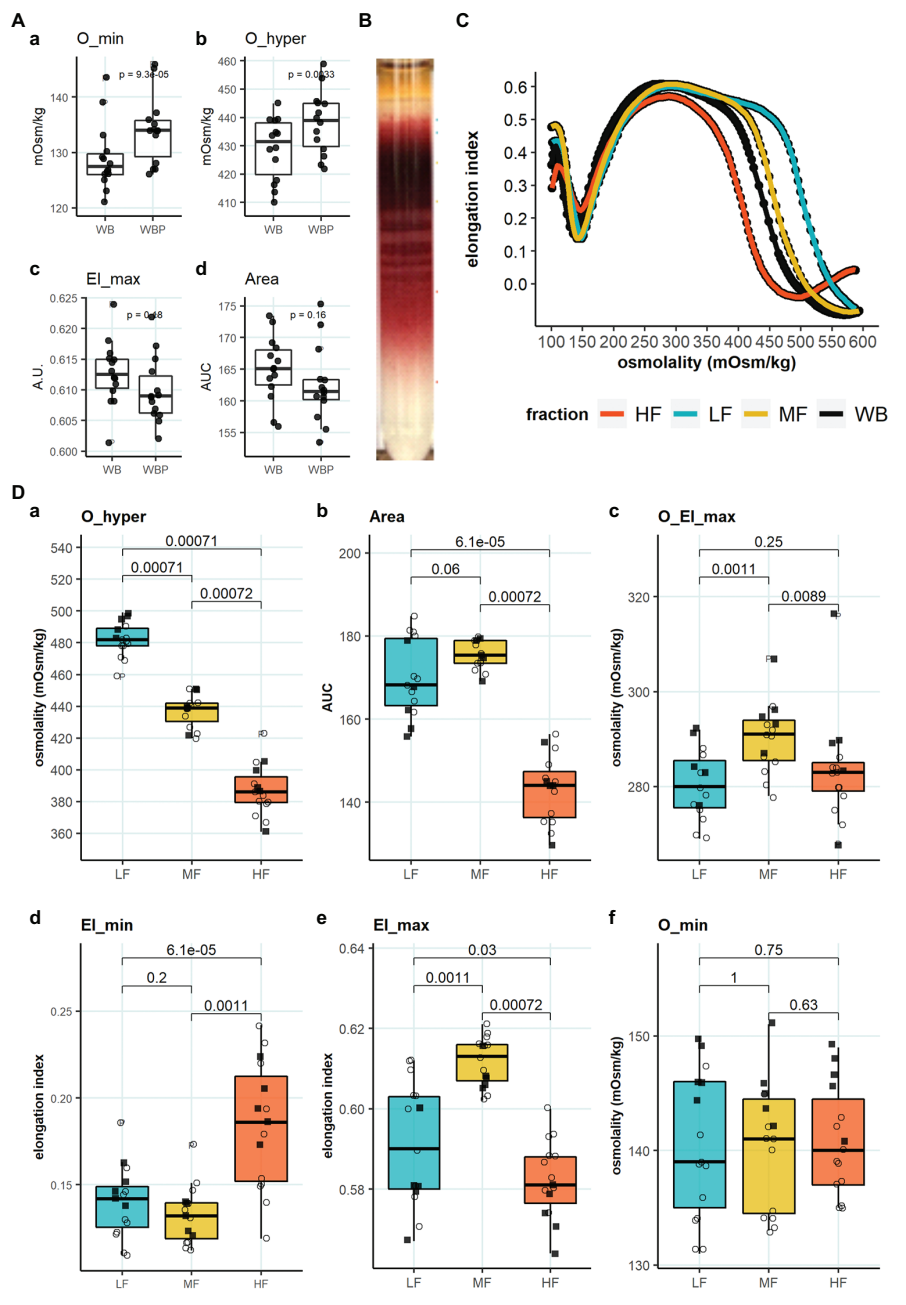
Lorrca indices of RBC subpopulations. **(A)** Influence of treatment with Percoll and centrifugation (WBP), paired *t* test, *N* = 14 **(B)** Representative image of Percoll density separation. **(C)** Representative osmoscan of whole blood (WB) and RBCs forming the L-, M-, and H-fraction **(D)** Lorrca indices of RBC subpopulations, paired wilcox test. a: O_hyper b: Area c: O_EI_max d: EI_min e: EI_max f: O_min. Females (circles) and males (squares); LF: low density fraction; MF: middle density fraction; HF: high density fraction; and EI: elongation index.

We have then tested the Lorrca indices for their possible association with the age of RBCs. Fractionation in Percoll density gradient was used to obtain L-, M-, and H fractions different in densities and in average age. In order to evaluate the possible impact of the fractionation procedure on RBC properties, osmoscans of whole blood were compared with those of whole blood that underwent all mock fractionation steps (centrifugation in Percoll gradient and washing steps) except for collection of fractions. Contact with Percoll and centrifugation procedures resulted in a right-ward shift of the osmoscan curve, and a concomitant increase in O_min (Figure 2Aa) and O_hyper (Figure 2Ab). All the findings indicated that fractionation was associated with overhydration of RBC. The experimental treatment had no influence on RBC deformability and the area under the curve (EI_max and Area; Figures 2Ac,Ad).

We then obtained the suspensions of cells forming L-, M-, and H-fractions (Figure 2B) and compared the osmoscan curves (Figure 2C) and Lorrca indices (Figure 2D) for these RBC subpopulations. O_hyper progressively decreased with an increase in RBC density (Figure 2Da). The osmoscan curve for the H-fraction was narrow compared to the curved for L- and M-fractions, indicating narrow range of tolerated osmolalities (Figure 2C). These changes were reflected by a decrease in the Area for the H-fraction (Figure 2Db). Both L- and H-fractions had their maximal deformability at osmolalities lower than the cells from the M-fraction (O_El_max; Figure 2Dc). The cells forming the H-fraction were more deformable at low osmolalities compared to the L- and M-fraction (EI_min; Figure 2Dd) but least deformable at isotonic osmolalities (EI_max; Figure 2De). RBCs forming the L-fraction were on average less deformable than those from M-fraction. The cells with low-, medium-, and high-density did not differ in their tolerance to hypoosmotic swelling (O_min; Figure 2Df).

### Characterization of RBCs Within L-, M-, and H-Fractions

**Figure 3:**
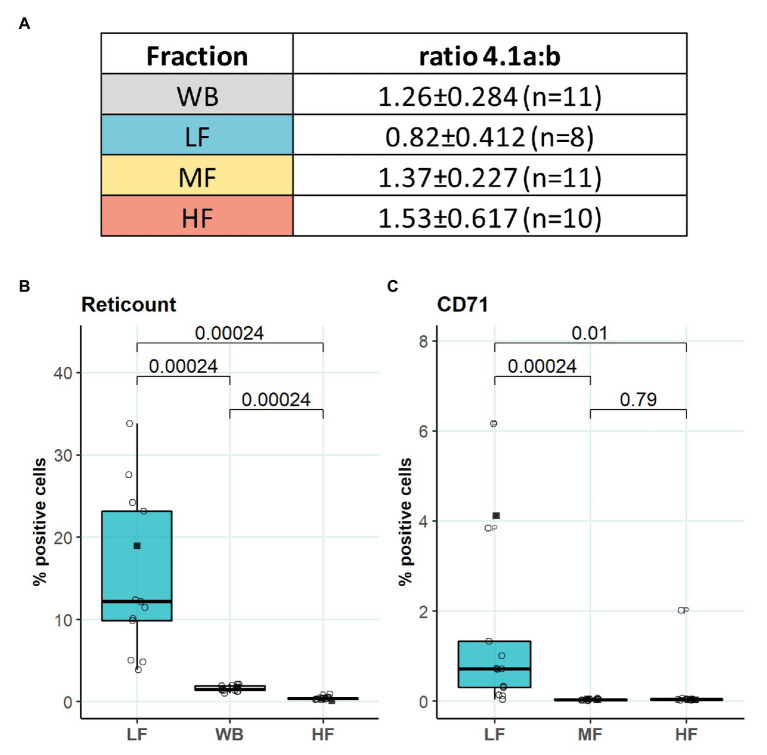
Average age of RBCs forming L-, M-, and H-fractions. **(A)** Band 4.1a:b ratio to determine the age of different fractions **(B)** RNA staining of L-, M-, and H-fraction, paired wilcox test, *N* = 13 **(C)** Transferrin receptor (CD71) staining of L-, M-, and H-fraction, paired wilcox test, *N* = 13. Females (circles) and males (squares); LF: low density fraction; MF: middle density fraction; HF: high density fraction, ratio 4.1a:b: the ratio between band 4.1a and 4.1b, CD71: transferrin receptor.

**Figure 4:**
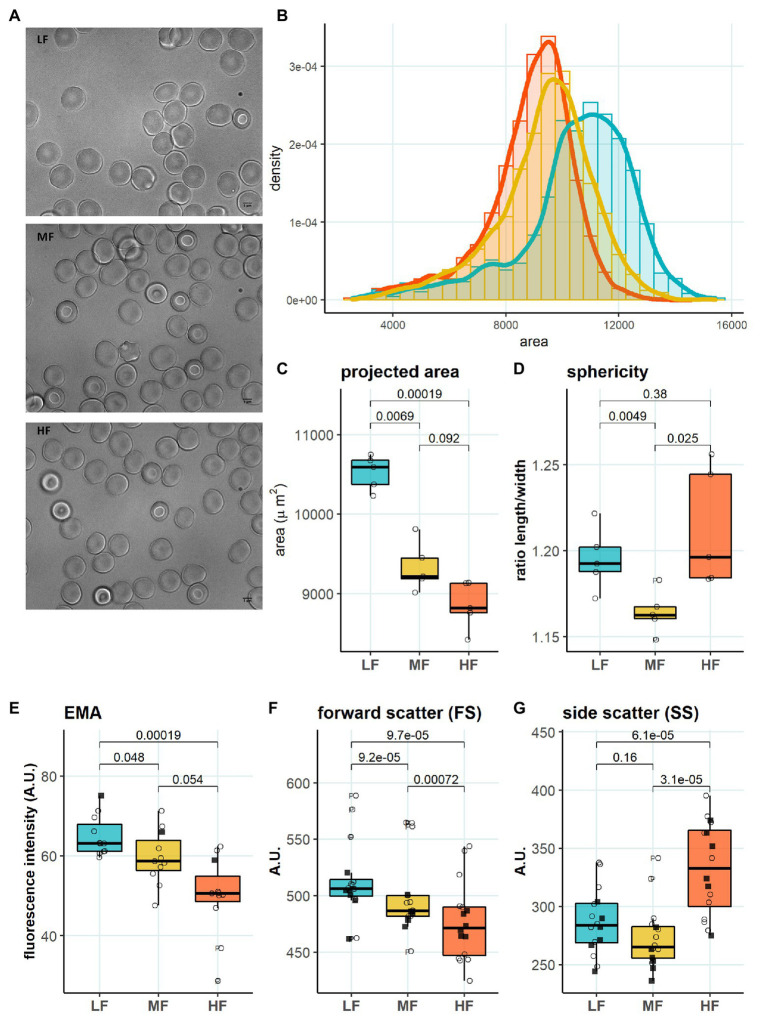
Morphology in RBC subpopulations. **(A)** Representative microscopy images of LF, MF, and HF **(B)** Histogram of projected area in different RBC subpopulations, binwidth = 500. **(C)** Projected area determined with Mask R-CNN, paired *t* test, *N* = 5 **(D)** Sphericity determined with Mask R-CNN, paired *t* test, *N* = 5 **(E)** EMA (band 3 protein) staining, paired *t* test, *N* = 11 **(F)** Forward scatter (FS), paired *t* test, *N* = 16. **(G)** Side scatter (SS), paired wilcox test, *N* = 16. Females (circles) and males (squares); LF: low density fraction; MF: middle density fraction; HF: high density fraction; and EMA: eosin-5-maleimide.

**Figure 5:**
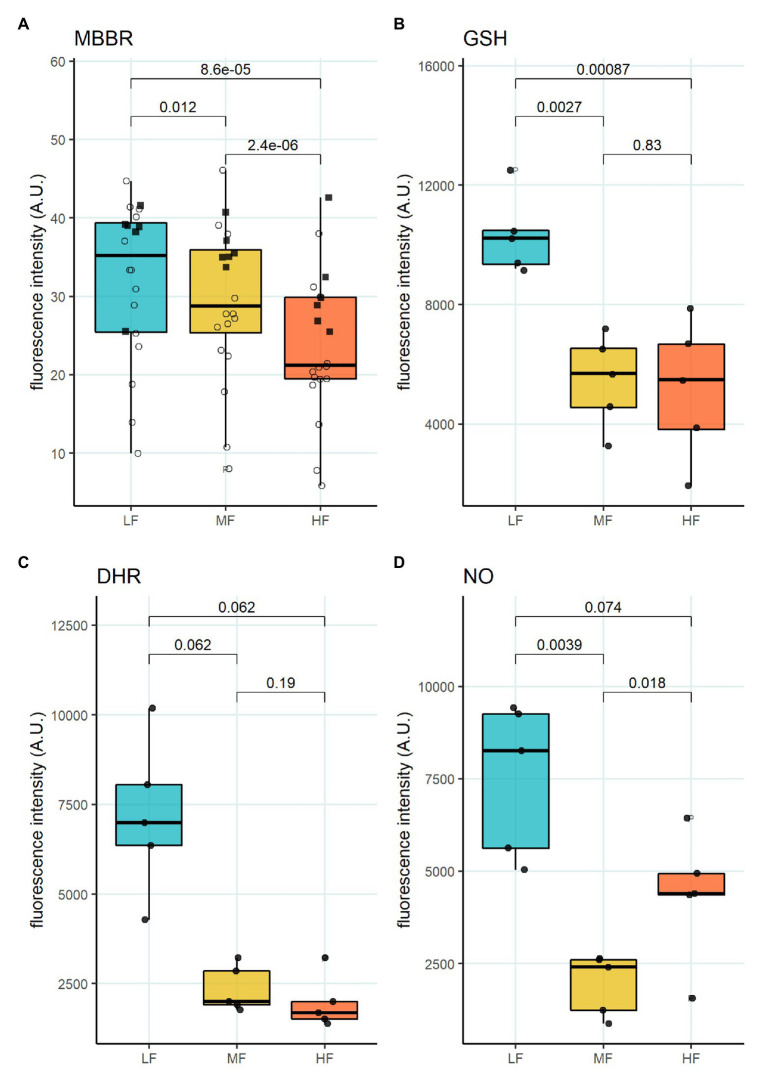
Oxidative state of RBC subpopulations. **(A)** mBBr staining, paired *t*-test, *N* = 20 **(B)** GSH staining with Thiol tracker violet, paired *t*-test *N* = 5 **(C)** DHR staining for H_2_O_2_ and ONOO^−^, paired wilcox test, *N* = 5 **(D)** DAF-DA staining for NO, paired *t*-test, *N* = 5. Females (circles) and males (squares); LF: low density fraction; MF: middle density fraction; HF: high density fraction; mBBr: monobromobimane; GSH: glutathione; DHR: dihydrodhodamine 123; and NO: nitric oxide.

We then aimed to identify the RBC age-dependent parameters other than density that could cause the differences in Lorrca indices for the RBC fractions. Those included the average age of cells forming the fractions, membrane surface area and morphological characteristics, and markers of redox state, and NO production. We have first proven that the cells forming L-, M-, and H-fractions differ from each other age-wise using 4.1a:b ratio and reticulocyte counts (Figures 3A,B). The cells forming the L-fraction were younger than those of the M-fraction, whereas the cells from H-fraction were the oldest of all as followed from the band 4.1a:b ratio (Figure 3A). Most of the reticulocytes (RNA-positive cells; Figure 3B) as well as immature ones positive for transferrin receptor (CD71; Figure 3C) were found within the L-fraction. Representative images of the cells forming the L-, M-, and H-fractions and the histograms of distribution of projected areas are shown in Figures 4A,B. Analysis of 940–1928 cells per fraction revealed that the cells of the L-fraction had the biggest projected area while the difference between M- and H-fraction was markedly less (Figure 4C). Analysis of projected area eccentricity revealed that morphology of the cells of the M-fraction was closer to circular (the longest to the shortest diameters’ ratio closer to 1), whereas cells of the L- and H-fractions were more ovaloid (sphericity; Figure 4D). In agreement with these observations, cells within the H-fraction showed the strongest EMA staining and the senescent high-density cells had the lowest EMA fluorescence intensity (Figure 4E), indicating membrane surface loss in this fraction. An indirect observation from FS, which characterizes the relative size of cells, revealed that RBC projected area in flow was progressively decreasing with an increase in cell density (Figure 4F). Furthermore, the cells forming the H-fraction had higher SS, which indicates the irregular, most likely echinocytic, shape of the cells (Figure 4G). Measurement of redox state was performed for the RBCs within L-, M-, and H-fractions by means of flow cytometry. mBBr staining was used to track protein and non-protein reduced thiols (Figure 5A), whereas Thiol tracker stained presumably glutathione (GSH; Figure 5B). Oxidative load was visualized using DHR staining (Figure 5C) and DAF-DA used for the detection of free radical and N_2_O_3_ levels in RBCs of L-, M-, and H-fraction (Figure 5D). The abundance of reduced thiols declined progressively with an increase in density (Figures 5A,B). However, it did not mirror the levels of oxidants (H_2_O_2_, HO^•^, and ONOO^−^) in the cells. Production of oxidants was maximal in the youngest cells of L-fraction and declined in mature and senescent cells (Figure 5C). NO levels were maximal in the L-fraction; surprisingly, it was followed by cells in the H-fraction in which NO production of the fluorescent dye was exceeding that in the M-fraction (Figure 5D).

## Discussion

This study revealed O_hyper as the parameter varying with the age of donors and responding to the changes in RBC age, and, hence, turnover. O_hyper is the parameter showing most variance for the healthy donors as follows from the previous studies (e.g., Fermo et al., 2017). It is amazing that age-dependence, but not gender-dependence of this parameter is resolved already within a pilot study. So far, only neonates had their own reference values for mean cellular volume (MCV), but, since osmoscan requires 200 μl of blood, this assay is not commonly used for neonates. We saw a clear difference in O_hyper between the study participants in their aged from 20 to 50 (Figure 1C). Further multi-center study with more blood samples of study subjects with no signs of hematological pathologies and several Lorrca devices will be needed to create guidelines, where patients and healthy controls are age matched.

Furthermore, the association between the O_hyper and RDW suggests that an increase in O_hyper may be a sign of severity of pathology, but this also must be confirmed in a follow-up study (Figures 1C,D). The third important finding, that has a potential clinical implication, is an association of Lorrca indices with RBC age (Figure 2D), and, hence, with RBC turnover, which is an indicator of severity of hemolytic anemia.

### Age- and Gender-Dependence of RBC Properties

Age-dependence of rheological properties of RBCs has been reported before (Simmonds et al., 2013). Larger, better hydrated RBCs of neonates are substantially more deformable compared to the RBCs of adults (Linderkamp et al., 1986). Adolescents (14–22 years) retain somewhat more deformable cells compared to the adults (23–33 years; Tomschi et al., 2018b). An enhanced deformability of the elderly-RBCs was also described (Stauder and Thein, 2014; von Tempelhoff et al., 2016). An increase in EI_max in senior people could be attributed to higher MCV and lower cytosolic viscosity that follow anemia of the elderly, which is attributable for multiple causes such as nutritional deficiency or chronic diseases (Stauder and Thein, 2014). Aging was shown to upregulate proinflammatory cytokines, asymmetric levels of dimethyl arginine, malondialdehyde, cholesterol in plasma, and these changes were gender-dependent (Stauder and Thein, 2014; Campesi et al., 2016). All these factors may enhance RBC damage and induce premature clearance, therefore facilitating the production of new RBCs in the elderly people.

Lifestyle and physical activity interfere with the age-driven changes in deformability of RBCs. In healthy people, there is limited knowledge about what influences those Lorrca indices. Lack of sexual dimorphism in RBC deformability was reported for athletes, independent of the type of sport (Tomschi et al., 2018b). Sedentary premenopausal women, however, showed a higher red cell deformability compared to men (Kameneva et al., 1999).

While we could not confirm gender-dependence in EI_max (Supplementary Figure S1D), the others reported superior deformability of RBCs in females compared to that of males that was not dependent on the estradiol concentration in plasma of females (Grau et al., 2018). Better deformability of RBCs in adult females over that in males was proven by filtration (Gelmini et al., 1987). In addition, postmenopausal effects of RBC renewal after blood loss were reported (Gelmini et al., 1987; Karolkiewicz et al., 2009; Campesi et al., 2016). Tomschi et al. (2018a) reported no difference between adolescent females and males, with sexual dimorphism emerging in adulthood, female RBCs become more deformable than those of adult males. Among the possible causes of this dimorphism, Nemeth et al. (2010) suggests higher intracellular water content in RBCs of females that undergo faster turnover and hence are younger. Whether younger RBCs age indeed directly translates into better deformability, remains unclear as we did not find any linear correlation between the RBC age and EI_max. Therefore, it is doubtful to conclude that better deformability of RBCs solely depends on their younger age is suggested (Figure 2De). Spontaneous iron deficiency might affect deformability of female RBCs (Brandao et al., 2009). Further environmental stresses that are not always associated with pathology include hypoxia that increases variance in RBC shapes and triggers reduction in deformability (Patel et al., 2013; Ycas et al., 2015; Sunnetcioglu et al., 2018). In our study, RDW was closely related to the hydration state of RBCs (Figure 1D).

### Age of Cells and Age-Associated Parameters

It is generally accepted that aging and senescence of RBCs results in loss of deformability (Linderkamp and Meiselman, 1982; Sutera et al., 1985; Chasis et al., 1989; Lutz et al., 1992; Bor-Kucukatay et al., 2005; McNamee et al., 2019). Information on the changes in RBC deformability during transformation from reticulocytes to young and mature cells is controversial. Chasis et al. (1989) reported lower deformability in RBC fractions enriched with reticulocytes compared to that of mature cells using ektacytometry and pipette aspiration technique. Our findings are in line with this observation (Figure 2De). Reduced ability of immature RBC membrane to deform was shown to outbalance high membrane surface-to-volume ratio of young cells (Chasis et al., 1989). In addition, our observations suggest that the RBCs within the L-fraction are better hydrated, and superior in band 3 protein abundance (Figures 2Da, 4E). Maximal projected area is observed for most of the RBCs forming L-fraction, but not for all of them (Figure 4B). A fraction of the cells from L-fraction are presented with projected area as small as the smallest cells from the H-fraction (Figure 4B). This observation is in line with the findings of Lew and Tiffert on the density reversal of terminally senescent cells (Lew and Tiffert, 2013). Therefore, despite that the RBCs in the L-fraction are on average younger than those in other fractions, this fraction most likely consists of a mixture of very young and senescent cells.

Another important feature of RBCs in the L-fraction is their ability to produce high levels of prooxidants and NO at the same time (Figure 5D). This is evident from the DHR staining that was used as a marker of free radical production (Figure 5C). This fluorescent probe does not sense superoxide anions, but is highly sensitive to the production of H_2_O_2_, HO^•^, and ONOO^−^ (Henderson and Chappell, 1993; Crow, 1997). NO production seems to be an important factor impacting RBC deformability (Bor-Kucukatay et al., 2003, 2005; Grau et al., 2018). The resulting effect of NO depends on the presence of reduced thiols, and oxidants (Diederich et al., 2018). Two processes following an increase in NO production are S-nitrosylation and tyrosine nitration (Pacher et al., 2007). Cells within the L-fraction are clearly pre-disposed for tyrosine nitration as the level of ONOO^−^ may be high based on the DHR and DAF-DA fluorescent readouts. Tyrosine nitration of calmodulin, that is, promoted by ONOO^−^ may further enhance NO production even in the presence of 50 nM concentrations of Ca^2+^ (Porter et al., 2020). As the cells from the L-fraction were reported to contain more free Ca^2+^ than the RBCs from any other fraction (Makhro et al., 2013), stimulation of both eNOS (Kleinbongard et al., 2006; Makhro et al., 2010) and NADPH oxidases (George et al., 2013) may be achieved. Despite with high pro-oxidants production rate, reduced thiol levels measured using mBBr and Thiol tracker are maximal in the RBCs of the L-fraction suggesting superior antioxidative defense capacity of these, at least in part, young cells (Figures 5A,B). Several ion transporters in RBCs are redox-sensitive, including KCC and NMDA receptors, as well as Na, K- and Ca-ATPases (Adragna et al., 2004; Zaidi et al., 2009; Bogdanova et al., 2016; Nakamura and Lipton, 2016). S-glutathionylation and S-nitrosylation may affect the properties of cytoskeletal components (Giustarini et al., 2019). Moreover, the main protein of RBC, hemoglobin, is redox-sensitive. Hemoglobin may undergo S-nitrosylation (Jia et al., 1996; Chan et al., 1998; Gladwin et al., 2000) and S-glutathionylation (Craescu et al., 1986; Mawatari and Murakami, 2004); both modifications affect the properties of the protein. Oxidative damages to membrane proteins of RBC accumulate over time. Redox modifications of the main membrane proteins of erythrocytes are closely related to the accumulation of membrane hemoglobin (Rifkind and Nagababu, 2013; Mohanty et al., 2014). An increase in the proportion of membrane-bound Hb leads to the formation of a redox-active complex, which contributes to further development of oxidative stress (Mohanty et al., 2014; Rifkind et al., 2018). An untargeted omics study has been performed (D’Alessandro et al., 2013), and proteomic and metabolomic properties of RBC fractions were determined. The study revealed no proteomic differences between the fractions. However, metabolic changes occurring with aging of RBCs could produce oxidative stress and affect enzymes’ activities by post-translational modifications. The most recent study of the same group (D’Alessandro et al., 2020) showed lower propensity to hemolysis and higher GSH levels in stored blood of healthy donors of the age group 50^+^ compared to that below 30. This observation may link metabolic and redox changes with those making O_hyper dependent on the age of donors (Figure 1C). At present, we may only state that an increase in the cytosolic reduced thiols may result in a drop in maximal deformability, and the underlying processes await further investigation.

### Word of Caution and Limitations of This Study

The natural question on how our data compare with findings of the other groups using the first-generation ektacytometers is difficult to answer due to the variability of the procedures used so far. Several reports on higher deformability of RBCs forming the L-fraction compared to that in the M- and H-fractions are known (Lutz et al., 1992; Linderkamp et al., 1993). It appears impossible to compare the findings between the groups. In some studies, (Linderkamp and Meiselman, 1982; Linderkamp et al., 1993) blood was fractionated by centrifugation in plastic capillaries and cutting them to obtain L-, M-, and H-fractions. The other group sedimented RBCs by centrifugation and aspirated from the top of the sedimented pellet to get enrichment with young cells that was confirmed by reticulocyte counts (Chasis et al., 1989; Linderkamp et al., 1993). In some studies leucodepletion by passing through the cellulose column in the presence of EGTA was performed prior to fractionation on EGTA-containing Percoll density gradient (Lutz et al., 1992), whereas the other studies leucodepletion step was omitted (McNamee et al., 2019).

High speed centrifugation with or without contact with Percoll used for RBC fractionation was shown to impact RBC properties by causing swelling and the corresponding shift in O_min and O_hyper (Figures 2Aa,Ab; Wiegmann et al., 2017). While we did not see a decrease of EI_max (Figure 2Ac), the others have reported a drop in EI_max after centrifugation (Nemeth et al., 2015; Kiss et al., 2016; Wiegmann et al., 2017). These observations make us question the feasibility of translation of the properties reported for the age-matched RBC after fractionation to the *in vivo* conditions. Therefore, in our study we did not compare fresh WB with the findings for the fractionated RBCs.

In agreement with earlier reports (McNamee et al., 2019), we have observed the high sensitivity of senescent cells in the H-fraction to sublethal mechanical stimulation, particularly under hyperosmotic conditions. At high shear stress, the cells forming L-fraction were less deformable than mature cells and showed it at slightly lower osmolalities than the cells within the M-fraction. However, the cells from the L-fraction were more resistant to hyperosmotic stimulus compared to that in M- and H-fractions under sublethal mechanical stimulation. This does not mean that the same behavior will be observed at lower shear rate. In fact, McNamee et al. (2019) revealed no cell age-dependent differences in deformability at low (physiologically relevant) shear rates.

Differences in the composition of the incubation medium add to the variance in the reported findings. Most of the studies were performed using PBS without Ca^2+^ or in the presence of EGTA to produce isosmotic Percoll solution and to incubate and wash RBCs. We have supplemented the incubation media with Ca^2+^, amino acids (glutamate, glycine, and arginine to support activity of NMDA receptors and NO synthase), Zn^2+^, and protein (BSA) to mimic plasma composition and preserve physiological regulation of Ca^2+^ uptake and to support NO production.

## Outlook

This pilot study suggests that making reference ranges of Lorrca indices for healthy people age-dependent and taking into account possible correlations between the RBC turnover rate (reticulocyte count, hemolytic markers, and RBC count) and the indices could make the assessment of severity of hemolytic state by way of Lorrca indices more precise. As we have shown, osmoscan may provide indirect information on RBC age, morphology (both static and in flow), as well as on the redox state of RBCs. We invite our colleagues to join us in collecting information on the impact of donor age on Lorrca indices and RBC turnover to produce new guidelines for definition of corrected reference range values that may improve precision of interpretation of the osmoscans of patients with hereditary hemolytic anemias.

## Data Availability Statement

The raw data supporting the conclusions of this article will be made available by the authors, without undue reservation.

## Ethics Statement

Ethical approval was not provided for this study on human participants because the study was performed as a part of detection of the reference values of the clinical hematological laboratory of Cantonal Hospital Winterthur. This work does not require a special permit of the ethical commission. The patients/participants provided their written informed consent to participate in this study.

## Author Contributions

AC, SF, and AB planned the study. AB supervised the study. JG provided the samples. AC, SF, DL, EM, IP, and KD performed experiments. AC, SF, IP, and AS analyzed the data. All the authors discussed the findings. AC, SF, IP, and AB were writing the manuscript. All authors contributed to the article and approved the submitted version.

### Conflict of Interest

The authors declare that the research was conducted in the absence of any commercial or financial relationships that could be construed as a potential conflict of interest.

## Supplementary Material

Supplementary Figure S1 Impact of gender on Lorrca indices. **(A)** Influence of gender on Lorrca indices, unpaired *t* test, *N*(females) = 28, *N*(males) = 17. EI: elongation index; F: female; M: male; and mBBr: monobromobimane.

## References

[ref1] Adragna N. C. Di Fulvio M. Lauf P. K. (2004). Regulation of K-Cl cotransport: from function to genes. J. Membr. Biol. 201, 109–137. 10.1007/s00232-004-0695-6, PMID: 15711773

[ref2] Balcerczyk A. Soszynski M. Bartosz G. (2005). On the specificity of 4-amino-5-methylamino-2',7'-difluorofluorescein as a probe for nitric oxide. Free Radic. Biol. Med. 39, 327–335. 10.1016/j.freeradbiomed.2005.03.017, PMID: 15993331

[ref3] Ballas S. K. Connes P. Investigators of the Multicenter Study of Hydroxyurea in Sickle Cell Anemia (2018). Rheological properties of sickle erythrocytes in patients with sickle-cell anemia: the effect of hydroxyurea, fetal hemoglobin, and alpha-thalassemia. Eur. J. Haematol. 101, 798–803. 10.1111/ejh.13173, PMID: 30204261 PMC6224298

[ref4] Bessis M. (1977). Erythrocyte form and deformability for normal blood and some hereditary hemolytic anemias (author’s transl). Nouv. Rev. Fr. Hematol. Blood Cells 18, 75–94. PMID: 896459

[ref5] Bogdanova A. Petrushanko I. Y. Hernansanz-Agustin P. Martinez-Ruiz A. (2016). “Oxygen Sensing” by Na,K-ATPase: these miraculous thiols. Front. Physiol. 7:314. 10.3389/fphys.2016.00314, PMID: 27531981 PMC4970491

[ref6] Bor-Kucukatay M. Meiselman H. J. Baskurt O. K. (2005). Modulation of density-fractionated RBC deformability by nitric oxide. Clin. Hemorheol. Microcirc. 33, 363–367. PMID: 16317245

[ref7] Bor-Kucukatay M. Wenby R. B. Meiselman H. J. Baskurt O. K. (2003). Effects of nitric oxide on red blood cell deformability. Am. J. Physiol. Heart Circ. Physiol. 284, H1577–H1584. 10.1152/ajpheart.00665.2002, PMID: 12521942

[ref8] Bosch F. H. Werre J. M. Schipper L. Roerdinkholder-Stoelwinder B. Huls T. Willekens F. L.. (1994). Determinants of red blood cell deformability in relation to cell age. Eur. J. Haematol. 52, 35–41. 10.1111/j.1600-0609.1994.tb01282.x, PMID: 8299768

[ref9] Brandao M. M. Castro Mde L. Fontes A. Cesar C. L. Costa F. F. Saad S. T. (2009). Impaired red cell deformability in iron deficient subjects. Clin. Hemorheol. Microcirc. 43, 217–221. 10.3233/CH-2009-1211, PMID: 19847056

[ref10] Campesi I. Occhioni S. Tonolo G. Cherchi S. Basili S. Carru C.. (2016). Ageing/menopausal status in healthy women and ageing in healthy men differently affect cardiometabolic parameters. Int. J. Med. Sci. 13, 124–132. 10.7150/ijms.14163, PMID: 26941571 PMC4764779

[ref11] Chan N. L. Rogers P. H. Arnone A. (1998). Crystal structure of the S-nitroso form of liganded human hemoglobin. Biochemistry 37, 16459–16464. 10.1021/bi9816711, PMID: 9843411

[ref12] Chasis J. A. Prenant M. Leung A. Mohandas N. (1989). Membrane assembly and remodeling during reticulocyte maturation. Blood 74, 1112–1120. PMID: 2752157

[ref13] Ciepiela O. (2018). Old and new insights into the diagnosis of hereditary spherocytosis. Ann. Transl. Med. 6:339. 10.21037/atm.2018.07.35, PMID: 30306078 PMC6174190

[ref14] Clark M. R. Mohandas N. Feo C. Jacobs M. S. Shohet S. B. (1981). Separate mechanisms of deformability loss in ATP-depleted and Ca-loaded erythrocytes. J. Clin. Invest. 67, 531–539. 10.1172/JCI110063, PMID: 6780609 PMC370596

[ref15] Clark M. R. Mohandas N. Shohet S. B. (1980). Deformability of oxygenated irreversibly sickled cells. J. Clin. Invest. 65, 189–196. 10.1172/JCI109650, PMID: 7350198 PMC371354

[ref16] Clark M. R. Mohandas N. Shohet S. B. (1983). Osmotic gradient ektacytometry: comprehensive characterization of red cell volume and surface maintenance. Blood 61, 899–910. PMID: 6831052

[ref17] Craescu C. T. Poyart C. Schaeffer C. Garel M. C. Kister J. Beuzard Y. (1986). Covalent binding of glutathione to hemoglobin. II. Functional consequences and structural changes reflected in NMR spectra. J. Biol. Chem. 261, 14710–14716. PMID: 3771548

[ref18] Crow J. P. (1997). Dichlorodihydrofluorescein and dihydrorhodamine 123 are sensitive indicators of peroxynitrite in vitro: implications for intracellular measurement of reactive nitrogen and oxygen species. Nitric Oxide 1, 145–157. 10.1006/niox.1996.0113, PMID: 9701053

[ref19] D’alessandro A. Blasi B. D'amici G. M. Marrocco C. Zolla L. (2013). Red blood cell subpopulations in freshly drawn blood: application of proteomics and metabolomics to a decades-long biological issue. Blood Transfus. 11, 75–87. 10.2450/2012.0164-11, PMID: 22871816 PMC3557492

[ref20] D’alessandro A. Fu X. Kanias T. Reisz J. A. Culp-Hill R. Guo Y.. (2020). Donor sex, age and ethnicity impact stored red blood cell antioxidant metabolism through mechanisms in part explained by glucose 6-phosphate dehydrogenase levels and activity. Haematologica. 10.3324/haematol.2020.246603, PMID: [Epub ahead of print] 32241843 PMC8094095

[ref21] Da Costa L. Suner L. Galimand J. Bonnel A. Pascreau T. Couque N.. (2016). Diagnostic tool for red blood cell membrane disorders: assessment of a new generation ektacytometer. Blood Cells Mol. Dis. 56, 9–22. 10.1016/j.bcmd.2015.09.001, PMID: 26603718 PMC4811191

[ref22] Diederich L. Suvorava T. Sansone R. Keller T. C. S. Barbarino F. Sutton T. R.. (2018). On the effects of reactive oxygen species and nitric oxide on red blood cell deformability. Front. Physiol. 9:332. 10.3389/fphys.2018.00332, PMID: 29867516 PMC5958211

[ref23] Fermo E. Bogdanova A. Petkova-Kirova P. Zaninoni A. Marcello A. P. Makhro A.. (2017). ‘Gardos Channelopathy’: a variant of hereditary Stomatocytosis with complex molecular regulation. Sci. Rep. 7:1744. 10.1038/s41598-017-01591-w, PMID: 28496185 PMC5431847

[ref24] Gelmini G. Delsignore R. Coiro V. (1987). Evaluation of erythrocyte deformability in pre-menopausal and post-menopausal women. Maturitas 9, 275–281. 10.1016/0378-5122(87)90010-7, PMID: 3431478

[ref25] George A. Pushkaran S. Konstantinidis D. G. Koochaki S. Malik P. Mohandas N.. (2013). Erythrocyte NADPH oxidase activity modulated by Rac GTPases, PKC, and plasma cytokines contributes to oxidative stress in sickle cell disease. Blood 121, 2099–2107. 10.1182/blood-2012-07-441188, PMID: 23349388 PMC3596970

[ref26] Giustarini D. Dalle-Donne I. Milzani A. Braconi D. Santucci A. Rossi R. (2019). Membrane skeletal protein s-glutathionylation in human red blood cells as index of oxidative stress. Chem. Res. Toxicol. 32, 1096–1102. 10.1021/acs.chemrestox.8b00408, PMID: 30945548

[ref27] Gladwin M. T. Shelhamer J. H. Schechter A. N. Pease-Fye M. E. Waclawiw M. A. Panza J. A.. (2000). Role of circulating nitrite and S-nitrosohemoglobin in the regulation of regional blood flow in humans. Proc. Natl. Acad. Sci. U. S. A. 97, 11482–11487. 10.1073/pnas.97.21.11482, PMID: 11027349 PMC17226

[ref28] Grau M. Cremer J. M. Schmeichel S. Kunkel M. Bloch W. (2018). Comparisons of blood parameters, red blood cell deformability and circulating nitric oxide between males and females considering hormonal contraception: a longitudinal gender study. Front. Physiol. 9:1835. 10.3389/fphys.2018.01835, PMID: 30618840 PMC6305760

[ref29] He K. Gkioxari G. Dollar P. Girshick R. (2020). Mask R-CNN. IEEE Trans. Pattern Anal. Mach. Intell. 42, 386–397. 10.1109/TPAMI.2018.2844175, PMID: 29994331

[ref30] Henderson L. M. Chappell J. B. (1993). Dihydrorhodamine 123: a fluorescent probe for superoxide generation? Eur. J. Biochem. 217, 973–980. 10.1111/j.1432-1033.1993.tb18328.x, PMID: 8223655

[ref31] Heo Y. Jung H. Shin S. (2015). Osmotic deformability of erythrocytes at various shear stresses. Clin. Hemorheol. Microcirc. 59, 211–218. 10.3233/CH-131761, PMID: 24004549

[ref32] Huisjes R. Bogdanova A. van Solinge W. W. Schiffelers R. M. Kaestner L. van Wijk R. (2018). Squeezing for life—properties of red blood cell deformability. Front. Physiol. 9:656. 10.3389/fphys.2018.00656, PMID: 29910743 PMC5992676

[ref33] Jackson K. E. Spielmann T. Hanssen E. Adisa A. Separovic F. Dixon M. W.. (2007). Selective permeabilization of the host cell membrane of Plasmodium falciparum-infected red blood cells with streptolysin O and equinatoxin II. Biochem. J. 403, 167–175. 10.1042/BJ20061725, PMID: 17155936 PMC1828889

[ref34] Jia L. Bonaventura C. Bonaventura J. Stamler J. S. (1996). S-nitrosohaemoglobin: a dynamic activity of blood involved in vascular control. Nature 380, 221–226. 10.1038/380221a0, PMID: 8637569

[ref35] Kaestner L. Bianchi P. (2020). Trends in the development of diagnostic tools for red blood cell-related diseases and anemias. Front. Physiol. 11:387. 10.3389/fphys.2020.00387, PMID: 32528298 PMC7264400

[ref36] Kameneva M. V. Watach M. J. Borovetz H. S. (1999). Gender difference in rheologic properties of blood and risk of cardiovascular diseases. Clin. Hemorheol. Microcirc. 21, 357–363. PMID: 10711771

[ref37] Karolkiewicz J. Michalak E. Pospieszna B. Deskur-Smielecka E. Nowak A. Pilaczynska-Szczesniak L. (2009). Response of oxidative stress markers and antioxidant parameters to an 8-week aerobic physical activity program in healthy, postmenopausal women. Arch. Gerontol. Geriatr. 49, e67–e71. 10.1016/j.archger.2008.09.001, PMID: 18990458

[ref38] Kiss F. Toth E. Miszti-Blasius K. Nemeth N. (2016). The effect of centrifugation at various g force levels on rheological properties of rat, dog, pig and human red blood cells. Clin. Hemorheol. Microcirc. 62, 215–227. 10.3233/CH-151965, PMID: 26444597

[ref39] Kleinbongard P. Schulz R. Rassaf T. Lauer T. Dejam A. Jax T.. (2006). Red blood cells express a functional endothelial nitric oxide synthase. Blood 107, 2943–2951. 10.1182/blood-2005-10-3992, PMID: 16368881

[ref40] LaCelle P. L. (1970). Alteration of membrane deformability in hemolytic anemias. Semin. Hematol. 7, 355–371. PMID: 5473418

[ref41] Lazarova E. Gulbis B. Oirschot B. V. van Wijk R. (2017). Next-generation osmotic gradient ektacytometry for the diagnosis of hereditary spherocytosis: interlaboratory method validation and experience. Clin. Chem. Lab. Med. 55, 394–402. 10.1515/cclm-2016-0290, PMID: 27559691

[ref42] Lew V. L. Tiffert T. (2013). The terminal density reversal phenomenon of aging human red blood cells. Front. Physiol. 4:171. 10.3389/fphys.2013.00171, PMID: 23847547 PMC3705192

[ref43] Linderkamp O. Friederichs E. Meiselman H. J. (1993). Mechanical and geometrical properties of density-separated neonatal and adult erythrocytes. Pediatr. Res. 34, 688–693. 10.1203/00006450-199311000-00024, PMID: 8284111

[ref44] Linderkamp O. Meiselman H. J. (1982). Geometric, osmotic, and membrane mechanical properties of density-separated human red cells. Blood 59, 1121–1127. PMID: 7082818

[ref45] Linderkamp O. Nash G. B. Wu P. Y. Meiselman H. J. (1986). Deformability and intrinsic material properties of neonatal red blood cells. Blood 67, 1244–1250. 10.1182/blood.V67.5.1244.1244, PMID: 3697506

[ref46] Llaudet-Planas E. Vives-Corrons J. L. Rizzuto V. Gomez-Ramirez P. Sevilla Navarro J. Coll Sibina M. T.. (2018). Osmotic gradient ektacytometry: a valuable screening test for hereditary spherocytosis and other red blood cell membrane disorders. Int. J. Lab. Hematol. 40, 94–102. 10.1111/ijlh.12746, PMID: 29024480

[ref47] Lutz H. U. Stammler P. Fasler S. Ingold M. Fehr J. (1992). Density separation of human red blood cells on self forming Percoll gradients: correlation with cell age. Biochim. Biophys. Acta 1116, 1–10. 10.1016/0304-4165(92)90120-j, PMID: 1371700

[ref48] Makhro A. Hanggi P. Goede J. S. Wang J. Bruggemann A. Gassmann M.. (2013). N-methyl-D-aspartate receptors in human erythroid precursor cells and in circulating red blood cells contribute to the intracellular calcium regulation. Am. J. Physiol. Cell Physiol. 305, C1123–C1138. 10.1152/ajpcell.00031.2013, PMID: 24048732

[ref49] Makhro A. Kaestner L. Bogdanova A. (2017). NMDA receptor activity in circulating red blood cells: methods of detection. Methods Mol. Biol. 1677, 265–282. 10.1007/978-1-4939-7321-7_15, PMID: 28986879

[ref50] Makhro A. Wang J. Vogel J. Boldyrev A. A. Gassmann M. Kaestner L.. (2010). Functional NMDA receptors in rat erythrocytes. Am. J. Physiol. Cell Physiol. 298, C1315–C1325. 10.1152/ajpcell.00407.2009, PMID: 20457837

[ref51] Mandavilli B. S. Janes M. S. (2010). Detection of intracellular glutathione using ThiolTracker violet stain and fluorescence microscopy. Curr. Protoc. Cytom. 53, 9.35.1–9.35.8. 10.1002/0471142956.cy0935s53, PMID: 20578109

[ref52] Mawatari S. Murakami K. (2004). Different types of glutathionylation of hemoglobin can exist in intact erythrocytes. Arch. Biochem. Biophys. 421, 108–114. 10.1016/j.abb.2003.10.012, PMID: 14678791

[ref53] McNamee A. P. Richardson K. Horobin J. Kuck L. Simmonds M. J. (2019). Susceptibility of density-fractionated erythrocytes to subhaemolytic mechanical shear stress. Int. J. Artif. Organs 42, 151–157. 10.1177/0391398818790334, PMID: 30073884

[ref54] Mohandas N. Clark M. R. Feo C. Jacobs M. S. Shohet S. B. (1981). Factors that limit whole cell deformability in erythrocytes after calcium loading and ATP depletion. Prog. Clin. Biol. Res. 55, 423–437. PMID: 6794036

[ref55] Mohandas N. Clark M. R. Jacobs M. S. Groner W. Shohet S. B. (1980a). Ektacytometric analysis of factors regulating red cell deformability. Blood Cells 6, 329–334. PMID: 6156730

[ref56] Mohandas N. Clark M. R. Jacobs M. S. Shohet S. B. (1980b). Analysis of factors regulating erythrocyte deformability. J. Clin. Invest. 66, 563–573. 10.1172/JCI109888, PMID: 6156955 PMC371685

[ref57] Mohanty J. G. Nagababu E. Rifkind J. M. (2014). Red blood cell oxidative stress impairs oxygen delivery and induces red blood cell aging. Front. Physiol. 5:84. 10.3389/fphys.2014.00084, PMID: 24616707 PMC3937982

[ref58] Nakamura T. Lipton S. A. (2016). Protein S-nitrosylation as a therapeutic target for neurodegenerative diseases. Trends Pharmacol. Sci. 37, 73–84. 10.1016/j.tips.2015.10.002, PMID: 26707925 PMC4698225

[ref59] Nemeth N. Kiss F. Furka I. Miko I. (2010). Gender differences of blood rheological parameters in laboratory animals. Clin. Hemorheol. Microcirc. 45, 263–272. 10.3233/CH-2010-1303, PMID: 20675908

[ref60] Nemeth N. Kiss F. Miszti-Blasius K. (2015). Interpretation of osmotic gradient ektacytometry (osmoscan) data: a comparative study for methodological standards. Scand. J. Clin. Lab. Invest. 75, 213–222. 10.3109/00365513.2014.993695, PMID: 25594795

[ref61] Pacher P. Beckman J. S. Liaudet L. (2007). Nitric oxide and peroxynitrite in health and disease. Physiol. Rev. 87, 315–424. 10.1152/physrev.00029.2006, PMID: 17237348 PMC2248324

[ref62] Patel K. V. Mohanty J. G. Kanapuru B. Hesdorffer C. Ershler W. B. Rifkind J. M. (2013). Association of the red cell distribution width with red blood cell deformability. Adv. Exp. Med. Biol. 765, 211–216. 10.1007/978-1-4614-4989-8_29, PMID: 22879035 PMC5939938

[ref63] Porter J. J. Jang H. S. Haque M. M. Stuehr D. J. Mehl R. A. (2020). Tyrosine nitration on calmodulin enhances calcium-dependent association and activation of nitric-oxide synthase. J. Biol. Chem. 295, 2203–2211. 10.1074/jbc.RA119.010999, PMID: 31914408 PMC7039552

[ref64] Relevy H. Koshkaryev A. Manny N. Yedgar S. Barshtein G. (2008). Blood banking-induced alteration of red blood cell flow properties. Transfusion 48, 136–146. 10.1111/j.1537-2995.2007.01491.x, PMID: 17900281

[ref65] Rifkind J. M. Mohanty J. G. Nagababu E. Salgado M. T. Cao Z. (2018). Potential modulation of vascular function by nitric oxide and reactive oxygen species released from erythrocytes. Front. Physiol. 9:690. 10.3389/fphys.2018.00690, PMID: 29930515 PMC5999795

[ref66] Rifkind J. M. Nagababu E. (2013). Hemoglobin redox reactions and red blood cell aging. Antioxid. Redox Signal. 18, 2274–2283. 10.1089/ars.2012.4867, PMID: 23025272 PMC3638511

[ref67] Sadafi A. Koehler N. Makhro A. Bogdanova A. Navab N. Marr C.. (2019). “Multiclass deep active learning for detecting red blood cell subtypes in brightfield microscopy” in Medical image computing and computer assisted intervention – Miccai 2019. Vol. 11764. eds. Shen D. Liu T. Peters T. M. Staib L. H. Essert C. Zhou S.. (Heidelberg, Berlin: Springer-Verlag), 685–693.

[ref68] Simmonds M. J. Meiselman H. J. Baskurt O. K. (2013). Blood rheology and aging. J. Geriatr. Cardiol. 10, 291–301. 10.3969/j.issn.1671-5411.2013.03.010, PMID: 24133519 PMC3796705

[ref69] Stauder R. Thein S. L. (2014). Anemia in the elderly: clinical implications and new therapeutic concepts. Haematologica 99, 1127–1130. 10.3324/haematol.2014.109967, PMID: 24986873 PMC4077071

[ref70] Sunnetcioglu A. Gunbatar H. Yildiz H. (2018). Red cell distribution width and uric acid in patients with obstructive sleep apnea. Clin. Respir. J. 12, 1046–1052. 10.1111/crj.12626, PMID: 28296212

[ref71] Sutera S. P. Gardner R. A. Boylan C. W. Carroll G. L. Chang K. C. Marvel J. S.. (1985). Age-related changes in deformability of human erythrocytes. Blood 65, 275–282. 10.1182/blood.V65.2.275.275, PMID: 3967082

[ref72] Tillmann W. (1986). Reduced deformability of erythrocytes as a common denominator of hemolytic anemias. Wien. Med. Wochenschr. 136, 14–16. PMID: 3548086

[ref73] Tomschi F. Bizjak D. Bloch W. Latsch J. Predel H. G. Grau M. (2018a). Deformability of different red blood cell populations and viscosity of differently trained young men in response to intensive and moderate running. Clin. Hemorheol. Microcirc. 69, 503–514. 10.3233/CH-189202, PMID: 29710695

[ref74] Tomschi F. Bloch W. Grau M. (2018b). Impact of type of sport, gender and age on red blood cell deformability of elite athletes. Int. J. Sports Med. 39, 12–20. 10.1055/s-0043-119879, PMID: 29165733

[ref75] von Tempelhoff G. F. Schelkunov O. Demirhan A. Tsikouras P. Rath W. Velten E.. (2016). Correlation between blood rheological properties and red blood cell indices(MCH, MCV, MCHC) in healthy women. Clin. Hemorheol. Microcirc. 62, 45–54. 10.3233/CH-151944, PMID: 26410854

[ref76] Waugh R. E. Narla M. Jackson C. W. Mueller T. J. Suzuki T. Dale G. L. (1992). Rheologic properties of senescent erythrocytes: loss of surface area and volume with red blood cell age. Blood 79, 1351–1358. 10.1182/blood.V79.5.1351.1351, PMID: 1536958

[ref77] Wiegmann L. de Zelicourt D. A. Speer O. Muller A. Goede J. S. Seifert B.. (2017). Influence of standard laboratory procedures on measures of erythrocyte damage. Front. Physiol. 8:731. 10.3389/fphys.2017.00731, PMID: 29042854 PMC5632557

[ref78] Ycas J. W. Horrow J. C. Horne B. D. (2015). Persistent increase in red cell size distribution width after acute diseases: a biomarker of hypoxemia? Clin. Chim. Acta 448, 107–117. 10.1016/j.cca.2015.05.021, PMID: 26096256

[ref79] Zaidi A. Fernandes D. Bean J. L. Michaelis M. L. (2009). Effects of paraquat-induced oxidative stress on the neuronal plasma membrane Ca^2+^-ATPase. Free Radic. Biol. Med. 47, 1507–1514. 10.1016/j.freeradbiomed.2009.08.018, PMID: 19715754 PMC2789485

[ref80] Zaidi A. U. Buck S. Gadgeel M. Herrera-Martinez M. Mohan A. Johnson K.. (2020). Clinical diagnosis of red cell membrane disorders: comparison of osmotic gradient Ektacytometry and eosin Maleimide (EMA) fluorescence test for red cell band 3 (AE1, SLC4A1) content for clinical diagnosis. Front. Physiol. 11:636. 10.3389/fphys.2020.00636, PMID: 32636758 PMC7318840

[ref81] Zama D. Giulietti G. Muratore E. Andolfo I. Russo R. Iolascon A.. (2020). A novel PIEZO1 mutation in a patient with dehydrated hereditary stomatocytosis: a case report and a brief review of literature. Ital. J. Pediatr. 46:102. 10.1186/s13052-020-00864-x, PMID: 32703298 PMC7379360

[ref82] Zaninoni A. Fermo E. Vercellati C. Consonni D. Marcello A. P. Zanella A.. (2018). Use of Laser Assisted Optical Rotational Cell Analyzer (LoRRca MaxSis) in the diagnosis of RBC membrane disorders, enzyme defects, and congenital dyserythropoietic anemias: a Monocentric Study on 202 Patients. Front. Physiol. 9:451. 10.3389/fphys.2018.00451, PMID: 29755372 PMC5934481

