# Supplementary material for: Donor Age and Red Cell Age Contribute to the Variance in Lorrca Indices in Healthy Donors for Next Generation Ektacytometry: A Pilot Study
Source: Front Physiol. 2021 Mar 2;12:639722. doi: 10.3389/fphys.2021.639722 (PMC7960761; doi:10.3389/fphys.2021.639722)
Supplement: Supplementary file 1 [file Data_Sheet_1.pdf]

Supplementary Table 1. Power calculations

lm = linear model, RDW = red cell distribution width, MCHC = mean corpuscular hemoglobin concentration, EI = elongation index, CD71 = transferring receptor, EMA = eosin-5-maleimide, mBBr: monobromobimane, GSH: glutathione, DHR: dihydrodhodamine 123, DAF-DA: 4-amino-5-methylamino-2',7'-difluorofluorescein diacetate, NO: nitric oxide

**Power calculations**		
	**Parameter**	**Power (%)**
**Figure 1**		
C	lm (O_hyper ~ age+gender)	86.3
D	lm (O_hyper ~ RDW)	93.3
E	lm (O_hyper ~ intracellular water)	99.9
F	lm (O_hyper ~ MCHC)	36.3
**Figure 2A**		
a	O_min	12.4
b	O_hyper	12.9
c	EI_max	6.6
d	Area	5.8
**Figure 2D**		
a	O_hyper	99.9
b	Area	99.6
c	O_EI_max	18.1
d	EI_min	70.4
e	EI_max	90.2
f	O_min	5
**Figure 3**		
B	Reticount	92.6
C	CD71	26.4
**Figure 4**		
C	Projected area	77.8
D	Sphericity	24.8
E	EMA	60.7
F	Forward scatter	9.2
G	Side scatter	86.3
**Figure 5**		
A	MBBR	9.4
B	GSH	54.6
C	DHR staining	66.8
D	DAF-DA staining (NO)	57.1
