# Supplementary figures and images for: Donor Age and Red Cell Age Contribute to the Variance in Lorrca Indices in Healthy Donors for Next Generation Ektacytometry: A Pilot Study
Source: Front Physiol. 2021 Mar 2;12:639722. doi: 10.3389/fphys.2021.639722 (PMC7960761; doi:10.3389/fphys.2021.639722)

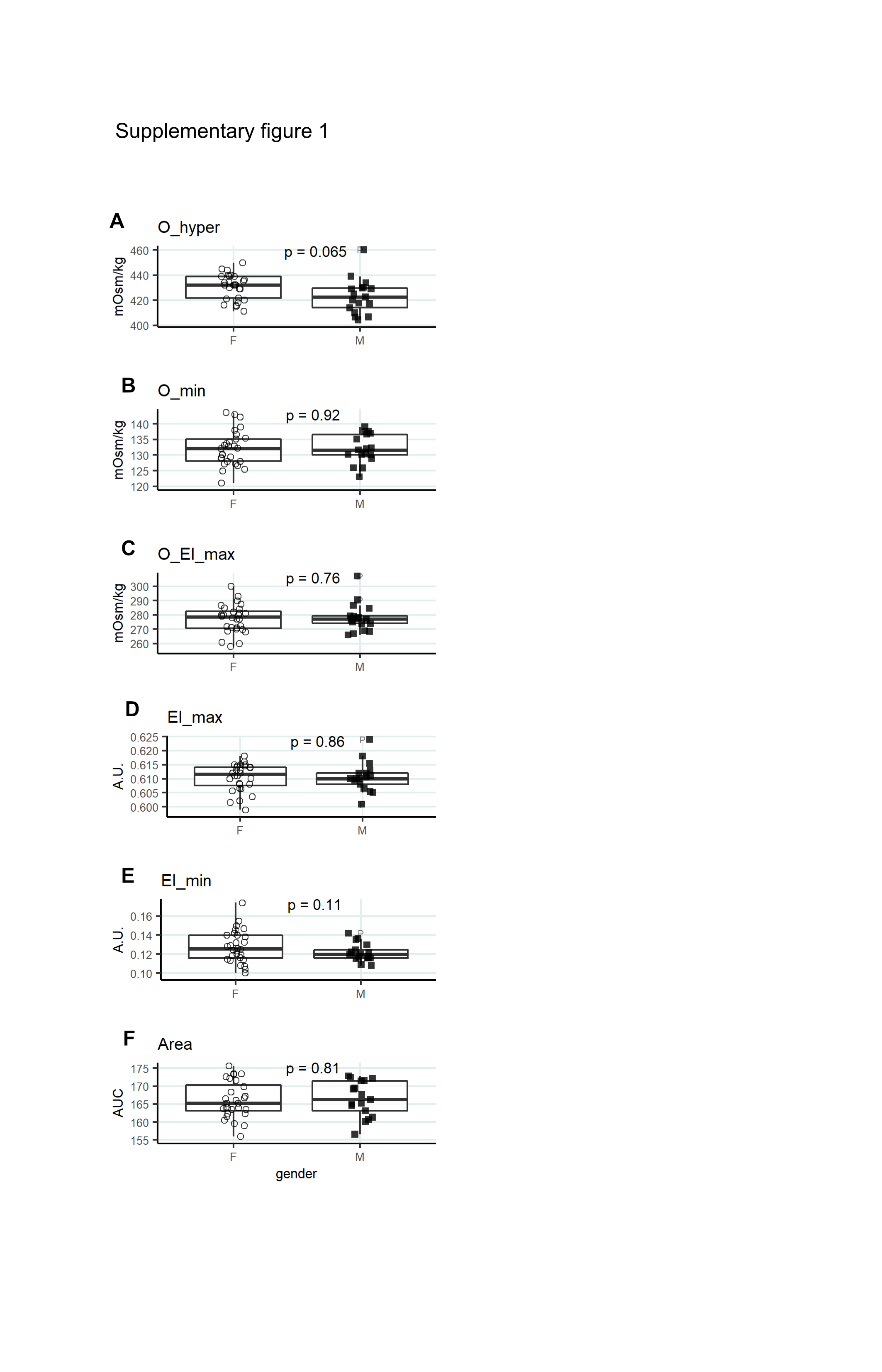

Supplement: Supplementary Figure S1 — Impact of gender on Lorrca indices. (A) Influence of gender on Lorrca indices, unpaired t test, N(females) = 28, N(males) = 17. EI: elongation index; F: female; M: male; and mBBr: monobromobimane. [file Image_1.tiff]
